# Superluminal Molecular and Nanomaterial Probes Based on Fast Ions or Electrons

**DOI:** 10.3390/molecules29122903

**Published:** 2024-06-19

**Authors:** Alexander Morrison, Vashista Muralidhara Srivatsa, Khashayar Ghandi

**Affiliations:** Department of Chemistry, University of Guelph, 50 Stone Road East, Guelph, ON N1G 2W1, Canada; amorri27@uoguelph.ca (A.M.); vmuralid@uoguelph.ca (V.M.S.)

**Keywords:** Cherenkov emissions, Cherenkov counting, Cherenkov spectroscopy, Cherenkov photometry

## Abstract

This work reviews the progression of chemical analysis via Cherenkov emissions, i.e., Cherenkov Photometry and Cherenkov Emission Spectroscopy, from its introduction in the literature up to modern developments. In presenting the history of this field, we aim to consolidate the literature, both for reference and contextualization. We present an argument aiming to untangle why this corner of research has seen little progress while so many other directly related aspects of Cherenkov research have flourished, as well as speak to the progress of the field in recent years and prospective direction in years to come.

## 1. Introduction

When a charged particle passes through a dielectric material having a velocity greater than the phase velocity of light in the medium, photons are emitted in a cone centered on the path of the particle. This pallid blue light is known as Cherenkov luminance. Despite being proposed as early as the 1880s by Heviside [1] and independently by Sommerfield [2] and Kelvin [3], at the turn of the 20th century, this work was largely ignored by the scientific community and forgotten until the experimental detection of the phenomenon by Cherenkov and Vavilov in the 1930s [4,5], followed swiftly by a robust theoretical description from Frank and Tamm [6,7,8,9]. For these latter efforts Cherenkov, Frank, and Tamm were awarded the Nobel Prize in Physics in 1954, and in the decades following, Cherenkov light has seen widespread adoption in a host of various basic science, industrial, and medical fields [10,11,12,13,14,15,16,17,18,19,20,21,22,23,24,25,26,27,28,29,30,31,32,33,34,35,36,37,38,39,40,41,42,43,44,45,46,47,48,49,50,51,52,53,54,55].

While a complete historical overview and sufficient theoretical treatment of this effect lie well outside the scope of this work (for a detailed overview of the history and theory of Cherenkov luminescence, we direct the reader to [27]), we would like to stress the degree to which technologies involving Cherenkov emissions have seen adoption. At this point in history, Cherenkov scintillation finds use in particle detection in essentially all technological spaces in which one might wish to detect particles, from biomedical applications [10,11], to high-energy accelerator applications [12], cosmic ray detection [13], neutron counting [14], and ICECUBE [15], wherein Cherenkov emissions produced in a km^3^ glacier in Antarctica are used to probe solar neutrinos. Cherenkov counting applications have a long history of use in various high-energy physics applications, the nuclear industry, and medical science, from detection of cosmic rays in the 1950s [16], to particle detection [17], radiation detection for environmental samples [18], and in vivo molecular imaging studies [19]. Indeed, in vivo Cherenkov imaging techniques [19,20] have seen 70 years of uninterrupted progress and use in medical applications. The list of widely adopted, groundbreaking, relevant work using Cherenkov emissions is massive.

During 70 years of progress and interest in Cherenkov research, the use of Cherenkov emissions as a chemical probe—a conceptually simple idea, and seriously proposing any sort of application of this effect—has seen slow progress, and, essentially, no interest or application. Considering the age and simplicity of the concept and how closely related it is to such mature research, the slow development is as odd as it is interesting. This article aims to take a historical perspective to gain insight into the slow development in the field and what we might learn about the future landscape of Cherenkov Spectroscopy. Additionally, we present recent developments in Cherenkov Spectroscopy, commenting on how these fit into the historical picture and what they might tell us about the trajectory of the field in the years to come.

Considering the above, we limit the discussion below largely to studies either directly measuring the spectral distribution of a Cherenkov signal produced by a solution under irradiation either from radionuclides in solution or an external source, or those concerned with determining the chemical character of a solution via Cherenkov emissions, i.e., Cherenkov Photometry or Cherenkov Emission Spectroscopy.

## 2. Historical Overview (1953–1985)

### 2.1. Early Considerations on the Spectral Character of Cherenkov Emissions

Consider a particle carrying a charge q, moving on a pathlength L through a dispersive dielectric medium of refractive index $n\left(\lambda \right)$ and permiability $\mu \left(\lambda \right)$. Should the particle carry a relative velocity $\beta =v/c_{\mathrm{vac}}$, such that nv>c, where c, $c_{\mathrm{vac}}$, and v are the phase speed of light in the medium, the speed of light in the vacuum, and the velocity of our particle, respectively, photons will be produced via the Cherenkov mechanism with a differential intensity $C\left(\lambda ,x\right)$ as described by the Frank–Tamm equation [9] (Equation (1)).

(1) $$C_{\;}\left(\lambda ,x\right)=\left(\frac{\lambda }{hc}\right)\int_{0_{\;}}^{L_{\;}}\frac{q^{2}}{4\pi }\mu \left(\lambda \right)\frac{c}{\lambda }\cdot \left(1-\frac{1}{\beta^{2}n{\left(\lambda \right)}^{2}}\right)dx.$$

Some fraction of these emissions is attenuated via scattering or absorption as they move through the medium. In the literature, this is commonly referred to as quenching the signal. By measuring the quenching of a Cherenkov signal passing through an optically transmissive sample, compared to some reference samples, material characteristics could in principle be extracted in a method like standard optical spectroscopy.

Although this has not been used for a long time, Cherenkov Photometry has a long history with arguably the first instance of use as an analytic technique to measure radionuclides performed by Belcher in 1952 [21], who measured the Cherenkov signal of various β-active radionuclides as detected by a photomultiplier. By comparing the Cherenkov signal to that of a continuous source of known intensity, they were able to determine the average energy of the β-particle by way of the integrated Frank–Tamm equation (Equation (1)), from which the specific radionuclide could be determined. While the study was principally concerned with using Cherenkov emissions to detect the presence of radionuclides, and not the chemical character of whatever they may be dispersed in, the author observed that the method fails in scattering solutions, e.g., those containing $UO_{2}^{2+}$. The potential for the Cherenkov signal as a spectroscopic probe was however not lost on Belcher, who wrote:

“The intensities of the effects measured by the photo-multiplier method were too feeble for spectrographic investigation. An attempt was made, … to record the emission from 100 mL of an aqueous solution of P 32 containing 200 μC/mL; … but even after an exposure of 10 days with the spectrograph slit wide open, the Cerenkov continuum was too feeble to be recorded, nor could any line spectrum be observed”.

[21]

This experiment was swiftly followed by Belcher and Anderson in 1953 [22], aiming to both demonstrate the potential of this technique in identifying the presence of a wider swath of radionuclides and to measure the Cherenkov spectral distribution of a Cherenkov signal in solution, as hinted at in Belcher’s previous work. This was achieved using the signal from a 7-day exposure to a “concentrated” (the actual concentration is not noted, but the specific activity of the sample (5.25 mc mL^−1^) was ~42 times greater than that of the P 32 solution) solution of ${\left(NH_{4}\right)}_{2}HPO_{4}$. The spectrum was observed to be featureless, in agreement with theory. What followed was a great deal of work aiming to leverage Cherenkov emissions to determine the radioactivity of a solution, i.e., Cherenkov counting. During this time, investigations regarding the medium effects on Cherenkov emissions were largely concerned with correcting for quenching to determine the average energy of the particle more accurately [23].

### 2.2. Cherenkov Photometry

The potential application of Cherenkov emission as an analytical tool, although as a measure of radiation by radionuclides, began in earnest a full decade after Belcher’s initial attempt by Haberer in 1965 [24], who demonstrated that the quenching of a Cherenkov signal produced by K 40 in solutions of picric acid was linear across a concentration range of 0.02–200 mg L^−1^. Additionally, Haberer’s study was the first published use of the term “Cherenkov Photometry”. This was followed shortly by Jordan and Köberle in 1969 [25], who determined that the quenching of the Cherenkov amplitudes of Sr 90 and Y90 radionuclides was a linear function of the concentration in solutions of $Na_{2}CrO_{4}$ over a range of 1–4 mg mL^−1^, and with this regression, they were able to determine the concentration of sodium chromate to within ~1% in this range.

**Figure 1:**
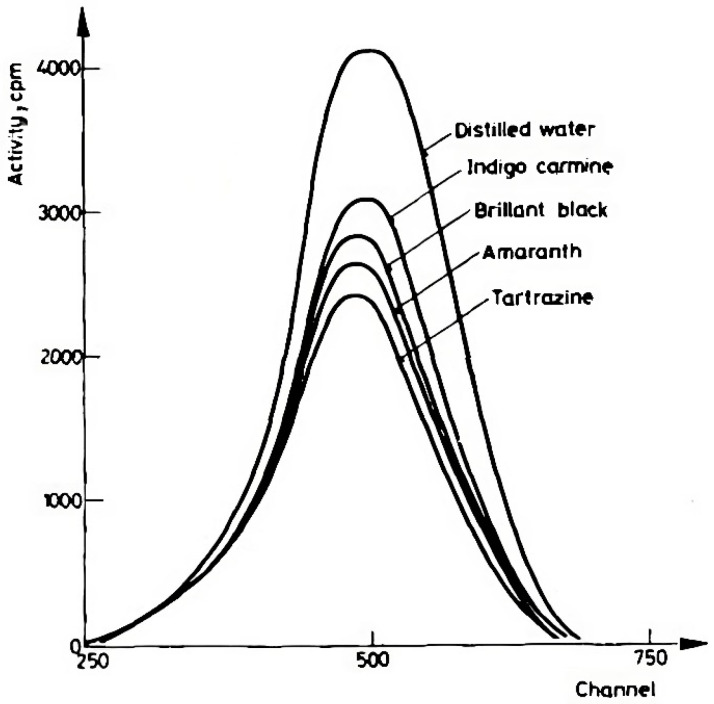
Cherenkov emission spectra of various dyes stimulated by an external source [27]. The figure was used with permission from the journal.

Over the next decade, work focused on Cherenkov emission spectra was carried out. Such work was concerned with peak amplitudes for detecting and characterizing radioisotopes in solutions. A clear example is the measurements reported in Francois’s 1973 work on the topic [26]. It was a full decade before Cherenkov Photometry would pick back up again, prompted by a series of studies from Kulcsár and coworkers, beginning in 1978. Kulcsár’s 1978 study [27] was on the concentration dependency of various water-soluble dyes on Cherenkov spectra. In the first study, solutions of Indigo carmine, Brilliant Black, Amaranth, and Tartrazine were placed in front of various standard emission sources containing aqueous solutions of K40, Cs137, or P32. The Cherenkov spectrum was recorded (Figure 1).

The concentration range was 1–10 ppm. The quenching coefficients of the dyes were determined from the linear relationship between the log of concentration and detection efficiency relative to that of water. Most materials absorb light in the spectrum of Cherenkov luminescence, which leads to lower intensity being observed in an optically dense medium. This is taken into account by considering a quenching coefficient. The study [27] was a turning point in Cherenkov Photometry, as the use of an external emission source decoupled the substance being probed from the radionuclide producing the emissions. Such a decoupling allows for Cherenkov emissions to be used as a probe light to investigate material.

**Figure 2:**
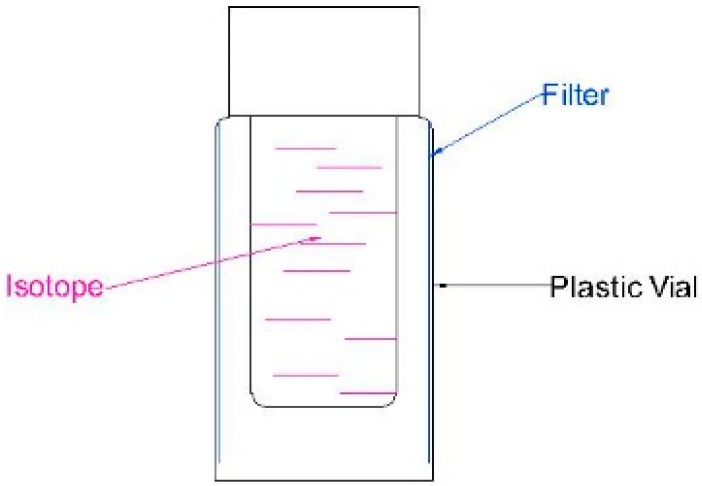
Schematic of the sample holder used in [29].

Kulcsár’s initial study was focused on working out a methodology for Cherenkov Photometry and demonstrating that the proposed methodology worked. As this was the case, the study employed water-soluble dyes with well-understood spectra properties, but little application or relevance outside their use as a standard. Kulcsár’s 1980 follow-up study [28] was somewhat more ambitious as it probed the linear/log relationship of the Cherenkov signal in solutions of chromate ions under irradiation from a much wider range of radionuclides. Kulcsár followed this with the publication of two papers in 1982. The first [29] demonstrated that by using a series of spectral filters around a sample (Figure 2), the spectra of individual radioisotopes could be more reliably distinguished. The second paper [30] used this technique to analyze and catalogue the spectra of a wide range of isotopes. The study marks the first application of Cherenkov Photometry.

Parallel to this work, González-Gómez and coworkers studied the colour-quenching effects of solutions on Cherenkov signals in counting experiments. They found the use of radionuclides dissolved in the solution to be a poor methodology and, in 1983, they proposed [31] that the use of a glass capillary housed in the sample as an emission source was a better solution. To demonstrate this, they performed statistical analysis on spectra of commercially available dyes produced by both the method of dissolution and that of their proposed capillary method. They concluded that the capillary method was superior. This appears to be the first instance where the statistical significance of Cherenkov Photometry data was considered and applied to measure the concentration of radionuclides.

In 1985, in a study [32] on Cherenkov emissions in optically transmissive media, Swailem and Moussa presented a series of emission spectra produced by a handful of emission sources in unspecified “different transparent media”. Based on the results, the authors state:

“These curves show that there is no significant difference in the shapes of Cerenkov spectra for the different radioisotopes in the different media.”

In 1990, Attas et al. [33] proposed using Cherenkov emissions to verify declared inventories of used nuclear fuel stored in water bays via amplification of the Cherenkov signal produced by the fuel assemblies. Attas et al. measured the percentage transmission of Cherenkov emissions at relative intensities, which shed light on the degree to which solar blind filters serve to detect Cherenkov emissions from used nuclear fuel kept underwater. The comparison of these curves showed the percentage transmission of Cherenkov luminescence per relative intensity, which is the amount of Cherenkov emissions that go through the solar blind filter. Greater overlap at a specific wavelength resulted in increased transmission of Cherenkov radiation through the filter at that wavelength. The study demonstrated that the solar blind filter enabled the detection of Cherenkov emissions throughout the wavelength range of 230 nm to 360 nm. At 300 nm, the filter had the best transmission percentage, and at 280 nm, it had the greatest overlap. At this point, it appears that research into spectroscopic studies on Cherenkov dried up again, which was not a logical move when we look back at the history, considering the great potential of Cerenkov radiation in many applications.

### 2.3. Cherenkov Emission Spectroscopy

#### 2.3.1. Probing Oxygen Concentration in Cells

Research on Cherenkov Photometry in the 2010s has been dominated by using Cherenkov radiation in tissue to stimulate fluorescent or phosphorescent compounds which act as a biomarker to image tumours. While not the focus of this review per se examples of such studies are referenced here [19,34,35,36,37,38,39,40,41].

The conversation regarding spectroscopic properties of solutes under irradiation began again in 2012, with a study by Glaser et al. [42], who showed that the degree of oxygen saturation in blood could be distinguished in simulated tissue via Cherenkov luminance. The study investigated the intensity of the Cherenkov spectrum at different wavelengths with no blood, oxygenated blood, and deoxygenated blood. Deoxygenated blood had a higher intensity before 560 nm, while oxygenated blood had a higher intensity between 560 nm and 580 nm, decreasing again between 580 nm and 600 nm. Oxygenated blood consistently exhibited higher intensity than deoxygenated blood between 600 and 800 nm. Axelsson et al. [43] expanded on this, demonstrating that Cherenkov emissions could quantitatively determine the hemoglobin concentration again in simulated tissue. This work was expanded upon by Zhang et al. [44] later that year. They showed that the degree of oxygenation could be determined in vivo in rats by exposing both live and dead rats to electron beams. In 2012, this work was further expanded upon by the same group [44], who probed the tissue depth dependency of Cherenkov signals, again in simulated tissues, to substantiate the usefulness of this technique in actual medical trials. At this point, the conversation on Cherenkov Spectroscopy as a medical tool shifted towards detecting the emissions of phosphors implanted in tumour cells excited by Cherenkov. This may have been due to the characteristically blue Cherenkov glow being poorly suited for transmission in tissue, which, of course, strongly scatters blue light. Techniques that use this glow to excite intermediates that re-emit in a more favourable longer wavelength band are preferable within this context.

#### 2.3.2. Systems of Nanoparticles and Beyond

**Figure 3:**
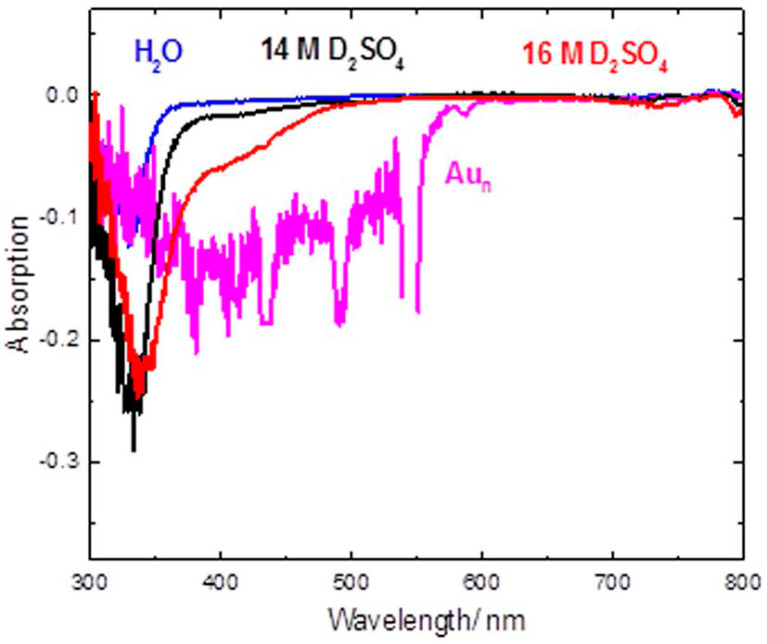
Cherenkov signals in “conventional” dielectric solutions (blue, black, red) as well as in a dilute solution of gold nanoparticles (pink). Note that negative absorption is interpreted as emission in the study [45]. Reproduced under the terms of the CC-BY 4.0 license. [45] Copyright 2018, Ghandi, K., published by Springer.

Lastly, we note the recent work by Ghandi et al. in 2019 [45]. This work demonstrated (Figure 3) that extremely dilute (particle concentration = 3.3 × 10^−8^ M) aqueous solutions of gold nanoparticles exhibit strong Cherenkov emissions. The study suggested that this potentially could lay the way for Cherenkov spectroscopic techniques to be applied within the nuclear industry. Deposition of bulk metals—presumably beginning as metal nanoparticles in coolant streams—may be detected. These spectra were interpreted as negative absorbance spectra using a pulse radiolysis setup once the contributions of the laser light had been subtracted. Given the orders of magnitudes of difference in luminosity of the two light sources, the subtracted data were necessarily somewhat noisy. This was compounded by the strongly scattering nature of solutions of gold nanoparticles. As such, Cherenkov measurements of this system taken in this way necessarily exhibited large errors. While the observation was predictably noisy, the presence of a large signal was unarguable, and the presence of some features was potentially observed. As such, follow-up studies were needed to pin down the exact degree to which this “enhancement” would have spectroscopic potential.

**Figure 4:**
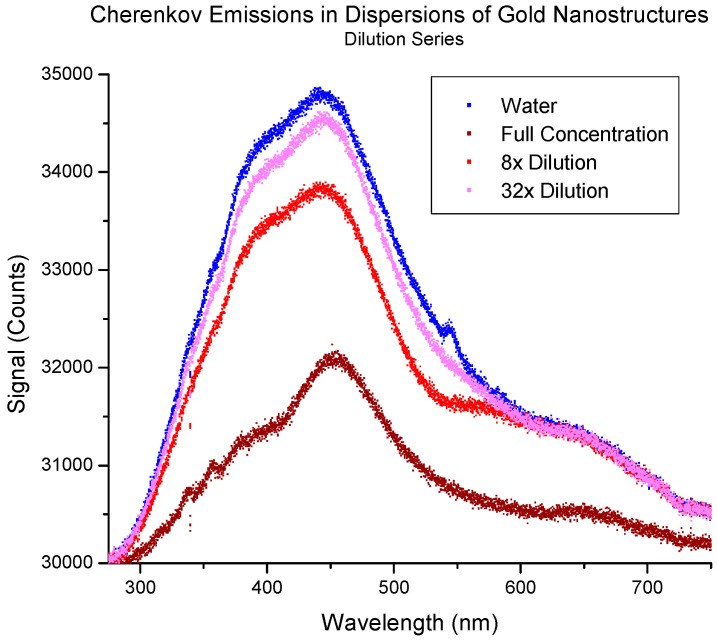
Cherenkov emission spectra for various dilutions of ~40 nm gold nanoparticles. Note the absence of any enhancement relative to water (blue) [46]. The full concentration was 100 pM. The figure was used with permission from the journal.

The aforementioned study was expanded upon with a series of experiments. Concentration series on various sizes of aqueous solutions of gold nanoparticles were probed with an electron beam and their Cherenkov response was recorded [46]. The study substantiated the observations of Cherenkov “enhancement” in certain systems of gold nanoparticles (Figure 4).

It was determined for the first time, via Mie Scattering Theory, that by correcting for attenuation of emitted photons, all gold nanoparticle systems will exhibit enhancement over water because of interactions between the medium and the Localized Surface Plasmon Resonance (LSPR) frequency—the resonant frequency of delocalized surface electrons. Theoretically, this LSPR response should not trend in size or concentration in the same order as the absorptivity of the LSPR. This suggests that the interaction between the Cherenkov effects and scattering effects may manifest as the overproduction or underproduction of photons relative to the Cherenkov signal in water. This was experimentally observed, where solutions of ~80 nm gold nanoparticles seemed to exhibit both an enhancement in and a quenching of the Cherenkov signal depending on concentration and which frequency one measured.

**Figure 5:**
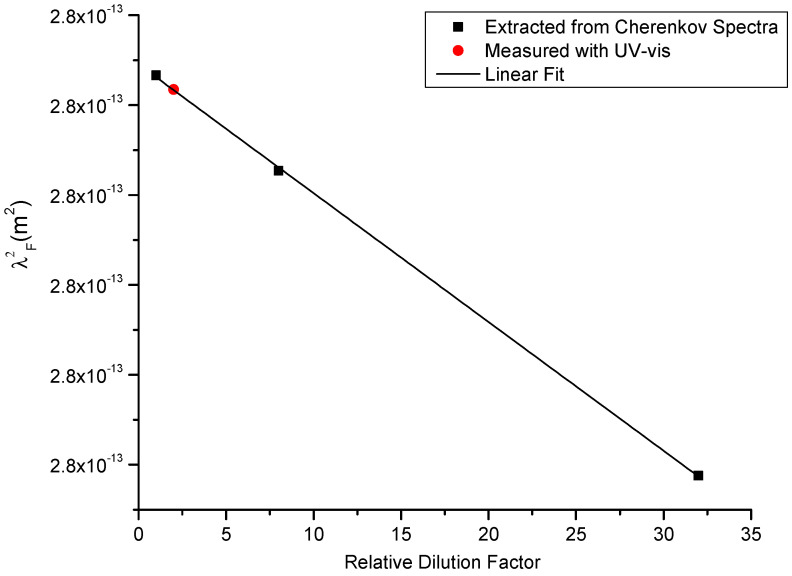
The linear trend in the square of extracted LSPR wavelengths. Black was extracted via Cherenkov Spectroscopy; red was measured with UV-vis spectroscopy (data from [46]).

Additionally, the study predicted that the previously mentioned mismatch between the character of photon emissions because of the interactions between LSPR and the attenuation of photons at the same wavelength would allow for the wavelength corresponding to the LSPR to be extracted uniquely from the Cherenkov spectra. Furthermore, the study considered dilutions of the same parent batch of nanoparticles using the relationship between the LSPR and the Surface Plasmon Resonance frequency, which is quadratic in relative dilution (Figure 5). The extracted wavelengths were shown to be quadratic as predicted as well as in trend with the same information extracted by UV-vis spectroscopy, a characterization technique ubiquitous in the literature. With this information, both materials, by way of LSPR and the concentration of the materials, by way of Froelich, can be extracted strictly using Cherenkov emissions with no other information required. This contrasts with the above studies in simulated tissue, where a calibration curve was required.

**Figure 6:**
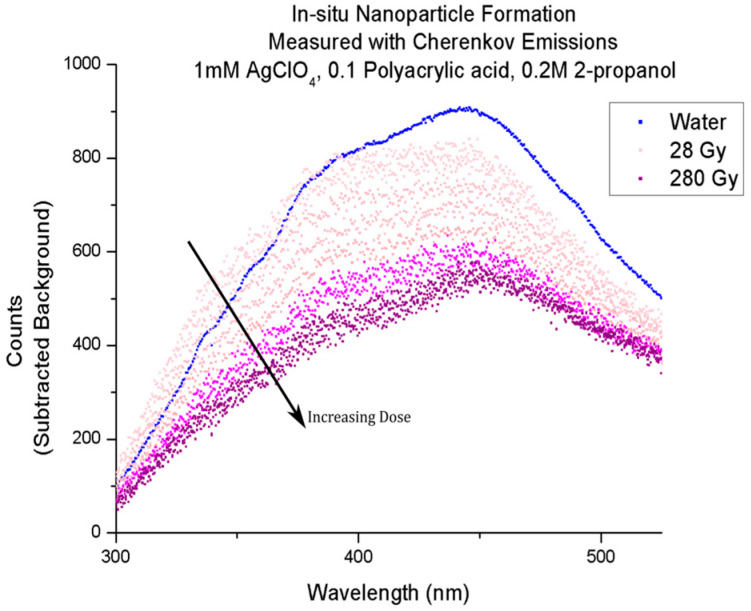
Progression of Cherenkov emissions in a solution of silver salt over iterative exposure to the electron beam. The result is consistent with silver being reduced to silver nanoparticles/clusters. Reproduced under terms of the CC-BY 4.0 license [47]. Copyright 2024, Ghandi, K., published by NRC Research Press.

**Figure 7:**
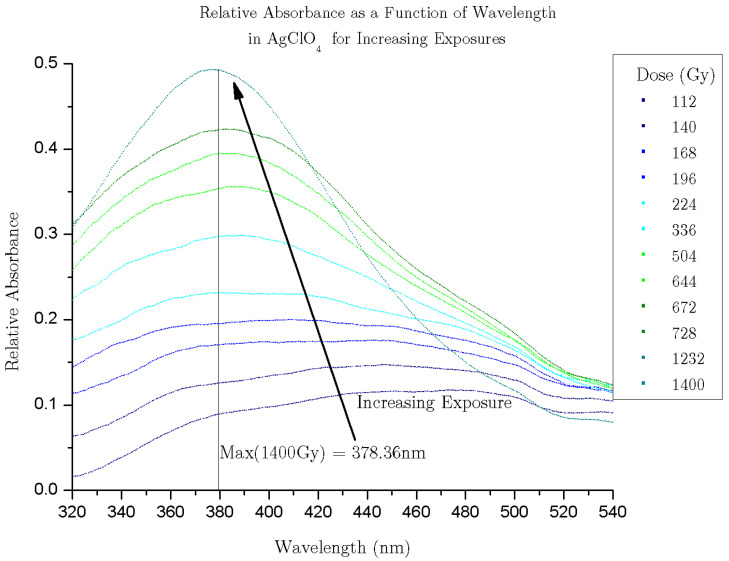
Cherenkov absorbance relative to water. Reproduced under terms of the CC-BY 4.0 license [47]. Copyright 2024, Ghandi, K., published by NRC Research Press.

A subsequent study was carried out in a solution of silver salt, measuring the change in the Cherenkov spectrum recorded (Figure 6 and Figure 7 (same data as Figure 6, smoothed and interpreted as absorbance relative to water because of a point source of light behind the sample. This analysis may not be robust enough to draw substantiated conclusions. Sufficient analysis requires consideration of no light at the far boundary and light being produced along the path of the electron, but it qualitatively shows the progression of the curves towards a “plasmon-like” absorption spectrum is qualitatively apparent)) of the solution exposed to iterative pulses of the electron beam [47]. The spectrum noticeably changed in a way consistent with the formation of silver nanoparticles via radiolytic reduction. The study demonstrated for the first time that the technique is not only sensitive to static material properties of solutions but can also be used to detect chemical changes in situ.

As a final note, measurements were made by the Ghandi group on many other industrially relevant materials both containing and in the absence of nanoparticles. Mature analysis has yet to be carried out on most systems, but a cursory glance suggests that Cherenkov spectroscopic measurements may be useful or interesting in a very wide assortment of systems and for probing chemistry in a variety of nuclear reactors.

## 3. Historical Perspective

In this section, we review the pioneering work performed by different groups in history. The first two subsections have subheadings named after the research group that performed the early work. The following section is the modern research on the topic.

### 3.1. Belcher

In reviewing the history of Cherenkov spectroscopic techniques, we are presented with a stepwise progression; short bursts of productivity and progress, usually carried out by a handful of individuals, promptly followed by about a decade of silence. This slow jerky progress is particularly jarring when we consider that in the background of this discussion, other aspects of Cherenkov’s research saw incredible development and application.

Cherenkov counting [14,23,31], in vivo Cherenkov imaging [19,20], and Cherenkov scintillators [48,49] are all at this point in history well-established, mature topics that see widespread adoption in research, industrial, and medical applications. Cherenkov spectroscopic techniques, on the other hand, were arguably not even developed to the point of being quantitative until this century. This is confounded by the conceptual simplicity of using Cherenkov emissions as a chemical probe: Belcher hints at the potential of Cherenkov emissions as a spectroscopic tool in his 1953 paper. To reiterate, this was the first paper proposing any application of Cherenkov emissions.

Belcher’s series of three papers from the early 1950s are representative of the trend in Cherenkov spectroscopic research that they kicked off. In his first paper, the central result that Cherenkov emissions characterize radionuclides was a wildly successful breakthrough, opening the door for a new method of radiation detection and characterization. The influence of the medium was considered, but largely in service of this result. The central result of Belcher’s follow-up paper, the feasibility of Cherenkov emissions from Sr 90 and Y90 radionuclides serving as a standard light source in biomedical applications, was a hugely relevant and applicable result and, again, a breakthrough. In that paper, the spectral distribution of Cherenkov emissions in solution was measured, but this result was tucked away near the end of the paper and was hardly central, with the discussion being limited to noting that the results agreed with theory and ideas of how one might correct for this quenching in Cherenkov counting applications. His next paper from later that year demonstrated the feasibility of in vivo Cherenkov applications in bone marrow, which again was a wildly applicable breakthrough of a result, kicking off the study of Cherenkov applications in biomedical research. In these papers, the spectral properties of Cherenkov emissions were a sideshow, discussed in service of a series of massively impactful, relevant results for in vivo imaging. This would remain the trend moving forward. Perhaps if one were aiming to study Cherenkov Emissions in a post-Belcher world, it would be difficult to justify looking into difficult-to-measure spectral properties of emissions with no clear application, which seemed to neatly agree with theory while new immediately impactful, relevant, and applicable work on other aspects of Cherenkov emissions were being produced.

### 3.2. KULCSAR and González-Gómez

Jumping a full quarter-century to the works of Kulcsár and González-Gómez at the turn of the 1980s, we see how sparse the research on Cherenkov Photometry was. Certainly, some work was carried out in the mid-1960s, including the coining of “Cherenkov Photometry” by Haberer in 1965.

We briefly restate the following:

Kulcsár’s first paper [27] was concerned with concretely determining the following:

(1) The extent to which the Beer–Lambert law is valid in Cherenkov Photometric experiments.
(2) The emission sources that are ideal for photometric experiments.
(3) The material such sources should be housed in (glass or plastic).

The 1980 follow-up [28] was concerned with the following:

(1) Extending the concentration ranges of data on the photometry of chromate solutions.
(2) Expanding the scope of which radiation sources should be used and their effect on quenching.

In 1983, González-Gómez [31] was again back to measuring systems of dyes to complete the following:

(1) Demonstrate an alternate setup for photometry experiments.
(2) Demonstrate via statistical analysis that this setup is superior to that of Kulcsár.

For the sake of fairness, we should note that these were not the first studies on the concentration dependence on Cherenkov amplitudes; the advantages of an analytical method governed by the Lambert law were discussed by Jordan in the previous decade. In addition, they were not the first to discuss the generality of emission sources; this had was performed by Belcher in 1953 (Belcher noted that his result “… supports the prediction that the distribution is independent of the energy of the exciting radiation” [11]).

The reality of the field of Cherenkov Chemical Analysis was that 30 years after Belcher’s initial proposal, the cutting-edge research included the following:

- Demonstrating the validity of the Lambert law.
- Using “toy” systems.
- Determining the fundamental methodology by which photometry experiments should be carried out.
- Whether glass or plastic should be used to house emission sources (which was not yet determined).
- The first rigorous consideration of the statistical significance of Cherenkov Photometric measurements.

The immaturity of this field at this point was clear.

As to why this may have been, we turn to the third entry [30] in Kulcsár’s series on Cherenkov Photometry from 1982. The study used coloured filters to accentuate the degree to which the small variation in quenching of Cherenkov signal amplitudes among various radioisotopes, allowing for the spectra of an unknown sample to be compared to a database of known spectra and thus identified. In performing this analysis, Kulcsár was able to distinguish double-labelled samples in his lab. The study is the first (again, in fairness we should note that this was proposed by Erlick and Parker in 1968 [28]) actual application of Cherenkov Photometry.

For context, at this point in history, in vivo biomedical Cherenkov imaging, Cherenkov counting, and Cherenkov scintillators have all been used in widespread industrial and research applications for the better part of two decades. Perhaps the most fitting way to describe the status of Cherenkov Spectroscopy in the late 20th century is to highlight the following statement by Swailem and Moussa in their extensive 1985 study on Cherenkov spectra in transparent media:

“These curves show that there is no significant difference in the shapes of Cerenkov spectra for the different radioisotopes in the different media.”

However, we maintain that it is myopic to suggest that there are no significant differences. Our work demonstrates that differences between these curves are substantial enough to be leveraged into a spectroscopic tool [45,46].

### 3.3. Modern Studies: Towards Clinical Application

In the last decade, we saw the conversation regarding spectroscopic properties of solutions under irradiation picked back up by [43], with studies showing the potential of using the Cherenkov signal in a material under irradiation to determine the percent saturation of oxygen in hemoglobin. Studies following this result have largely aimed to bring this measurement into clinical trials and, ultimately, into clinical practice. As such, research in this area has focused on increasingly biologically relevant studies on that same result. They progress from Cherenkov signals in tissue phantoms to those in actual tissues, to live mice, and proposed clinical trials. This progression, while promising, and a far cry from the scattered unfocused studies of the previous century, is by necessity focused, with a narrow scope.

In our view, the power, specificity, and usefulness of Cherenkov spectroscopic phenomena are far more general than just determining the degree of oxygen saturation in hemoglobin. What these studies do demonstrate is that given a feasible use case, there is room for the clinical or industrial impact of such a technique. That is, such studies provide credence and legitimacy to subsequent Cherenkov spectroscopic research, either focusing on one use case or more general inquiries aiming to determine novel possible use cases. We are beginning to see such novelties with the results, described in Section 2.3.2 [45,46].

## 4. Conclusions and Notes towards the Future

There are many reasons why Cherenkov Photometric and Spectroscopic research has seen comparatively slow growth. Certainly, the measurements are difficult, both to perform and interpret, but many other successful experimental methodologies are comparably finicky. Therefore, this does not account for the lapses in progress.

The fundamental issue is, and has always been, that there is no obvious application for a Cherenkov spectroscopic tool. Fundamentally, Cherenkov Spectroscopy and Photometry are complicated UV-vis spectroscopy experiments with noisier, subtler data. It is difficult to find a use case where conventional methods fail to be more realistic and reliable. As such, serious research into the field has been scattered and minor.

That said, the progression in the research seems to be nearing an inflection point. The spectroscopic research conducted in the last decade is a great deal more quantitative and rigorous than the research before it. Importantly, the work outlined is conceived with explicitly relevant applications in mind—applications where currently, no other spectroscopic methods are of use. This overcomes the main obstacle preventing the development of Cherenkov spectroscopic techniques. To highlight this, one can look at the progression of studies by Axelsson in the 2010s. We see that when presented with a feasible application, Cherenkov spectroscopic techniques can foster a great deal of attention and research.

On this note, we should state that studies within a medical context are constrained by the sparse existence of Cherenkov radiation. The cases where such light may be produced at all are quite specific and the signals are weak. Additionally, the blue glow characteristic of Cherenkov emissions is reasonably inconvenient in medical applications as tissue tends to absorb strongly and scatter light in this domain. Despite this limitation, it is possible that if optical fibres are placed near the point where radiation is focused during radiation therapy, Cherenkov radiation could provide in situ probing of chemical transformations triggered by radiation in radiation therapy. This would be valuable when radiosensitizers are used, particularly when those may involve nanomaterials or nanoclusters. The limit is less in a nuclear context. At many stages of the nuclear supply chain, from refinement to core conditions, waste streams, and waste containment, Cherenkov luminescence is unavoidable and currently unleveraged. It would be shocking if a potential use case for Cherenkov spectroscopic techniques did not exist somewhere along this line. Indeed, our group is developing a methodology to determine nanocluster and nanoparticle formation in situ in reactors to make changes to coolant chemistry, thus preventing any potential blockage in the piping or any other problem in reactors or during nuclear waste storage in different stages of storage.

Given the above, as well as the potential of the recent research carried out by novel work on nanoparticles and ionic clusters under irradiation [45,46,47] (both specifically to the nuclear industry as well as in generality of the technique), we are comfortable in predicting that the 2020s will see Cherenkov spectroscopic techniques pass from obscure ideas to ideas seriously considered for practical use. We are beginning to see this within a medical context, but we would predict that the same will be true within nuclear industrial contexts. To move towards actual applications, we believe that further studies in systems beyond simple systems of nanoparticles or systems containing more industrially relevant metals (e.g., steel or copper) should be undertaken.

## Data Availability

No new data were created.
